# Comparative analysis of gene expression between mice and humans in acetaminophen-induced liver injury by integrating bioinformatics analysis

**DOI:** 10.1186/s12920-024-01848-0

**Published:** 2024-03-28

**Authors:** Shanmin Zhao, Yan Feng, Jingyuan Zhang, Qianqian Zhang, Junyang Wang, Shufang Cui

**Affiliations:** Department of Laboratory Animal Sciences, School of Basic Medicine, Naval Medical University, NO. 800 Xiangyin Road, 200433 Shanghai, China

**Keywords:** Gene expression, Mouse and human, Acetaminophen-induced liver injury, Bioinformatics analysis

## Abstract

**Objective:**

Mice are routinely utilized as animal models of drug-induced liver injury (DILI), however, there are significant differences in the pathogenesis between mice and humans. This study aimed to compare gene expression between humans and mice in acetaminophen (APAP)-induced liver injury (AILI), and investigate the similarities and differences in biological processes between the two species.

**Methods:**

A pair of public datasets (GSE218879 and GSE120652) obtained from GEO were analyzed using “Limma” package in R language, and differentially expressed genes (DEGs) were identified, including co-expressed DEGs (co-DEGs) and specific-expressed DEGS (specific-DEGs). Analysis of Gene Set Enrichment Analysis (GSEA), Gene Ontology (GO) and Kyoto Encyclopedia of Genes and Genomes (KEGG) were performed analyses for specific-DEGs and co-DEGs. The co-DEGs were also used to construct transcription factor (TF)-gene network, gene-miRNA interactions network and protein-protein interaction (PPI) network for analyzing hub genes.

**Results:**

Mouse samples contained 1052 up-regulated genes and 1064 down-regulated genes, while human samples contained 1156 up-regulated genes and 1557 down-regulated genes. After taking the intersection between the DEGs, only 154 co-down-regulated and 89 co-up-regulated DEGs were identified, with a proportion of less than 10%. It was suggested that significant differences in gene expression between mice and humans in drug-induced liver injury. Mouse-specific-DEGs predominantly engaged in processes related to apoptosis and endoplasmic reticulum stress, while human-specific-DEGs were concentrated around catabolic process. Analysis of co-regulated genes reveals showed that they were mainly enriched in biosynthetic and metabolism-related processes. Then a PPI network which contains 189 nodes and 380 edges was constructed from the co-DEGs and two modules were obtained by Mcode. We screened out 10 hub genes by three algorithms of Degree, MCC and MNC, including *CYP7A1, LSS, SREBF1, FASN, CD44, SPP1, ITGAV, ANXA5, LGALS3* and *PDGFRA*. Besides, TFs such as *FOXC1*, *HINFP*, *NFKB1*, miRNAs like mir-744-5p, mir-335-5p, mir-149-3p, mir-218-5p, mir-10a-5p may be the key regulatory factors of hub genes.

**Conclusions:**

The DEGs of AILI mice models and those of patients were compared, and common biological processes were identified. The signaling pathways and hub genes in co-expression were identified between mice and humans through a series of bioinformatics analyses, which may be more valuable to reveal molecular mechanisms of AILI.

**Supplementary Information:**

The online version contains supplementary material available at 10.1186/s12920-024-01848-0.

## Introduction

Acetaminophen (APAP) is the most usually used analgesic and antipyretic drug around the world. More than 60 million Americans are estimated to take APAP-containing products on a weekly basis [1]. Although therapeutic range would be considered to be safe, its overdose could induce acute liver failure (ALF) and even death [2, 3]. According to reported statistics, APAP causes 46% of all ALF cases in the United States, and accounts for 40–70% of all ALF cases in Great Britain and Europe [4, 5]. APAP-induced liver injury (AILI) has become one of the leading causes of ALF, which even considered as a public health issue [6]. Therefore, it is crucial to study the pathogenesis in order to find the effective methods to treat AILI.

Due to regulatory and ethical concerns, it is not possible to enroll human subjects for an in-depth study of DILI. The mouse is considered to be a powerful tool in elucidating human physiology and pathophysiology of AILI. For example, Li et al. [7] showed that Brg1 might be involved in mediating APAP-induced liver injury, according to mouse models. The concentrations of fibroblast growth factor 21 (FGF21) were compared between mice and human, which was considered to be a promising biomarker of APAP-exposed livers [8]. Inhibition of Xbp 1 could enhance autophagy and decrease CYP2E1 expression that resulted in ameliorating AILI in C57BL/6J mice [9]. Insights from mouse models has been enriching our understanding on the pathogenesis of AILI. But we should recognize that the animal researches cannot provide a full prediction of human outcomes, due to differences in pathogenesis among different species. For instance, it has showed there is a weak correlation between animals and humans regarding absorption and metabolism [10]. This might result in the injury process progresses much faster in mice than in humans [11]. CYP2E1 activities and hepatocyte sensitivity of human and mouse were not found correlation either [12]. Therefore, toxicity presentations vary among species have attracted increasing research attention. Cross-species comparison of gene regulation at the molecular level could not only reveal the differential gene expression patterns, but also help understand their similarities through homologous genes in further [13].

In this study, we compared gene expression profiling data between mice and humans with AILI and healthy controls, in order to identificate the differentially expression genes (DEGs). Further analysis was make to screen co-DEGs of the two datasets, which give us hub genes with stronger direct correlation between humans and mice. Our findings of these hub genes could provide a more powerful reference for the study of human APAP-induced related mechanisms and therapeutic targets using mouse models. First comparison of DEGs from APAP-induced liver injury in mice to those from patients, and we identified the biological processes common to both mouse models and patients.

## Materials and methods

### Data source

Datasets with GEO accession numbers GSE218879 and GSE120652 were downloaded from the Gene Expression Omnibus (GEO) database (http://www.ncbi.nlm.nih.gov/geo/). GSE218879 contains gene expression data based on RNA-seq analysis from liver samples obtained from 3 mice (C57BL/6) following intraperitoneal injection with 500 mg/kg APAP and 3 mice untreated with APAP. In GSE120652, gene expression data from 3 patients with AILI and three healthy controls were analyzed using microarray analysis.

### Analysis of differential gene expression

**Fig. 1:**
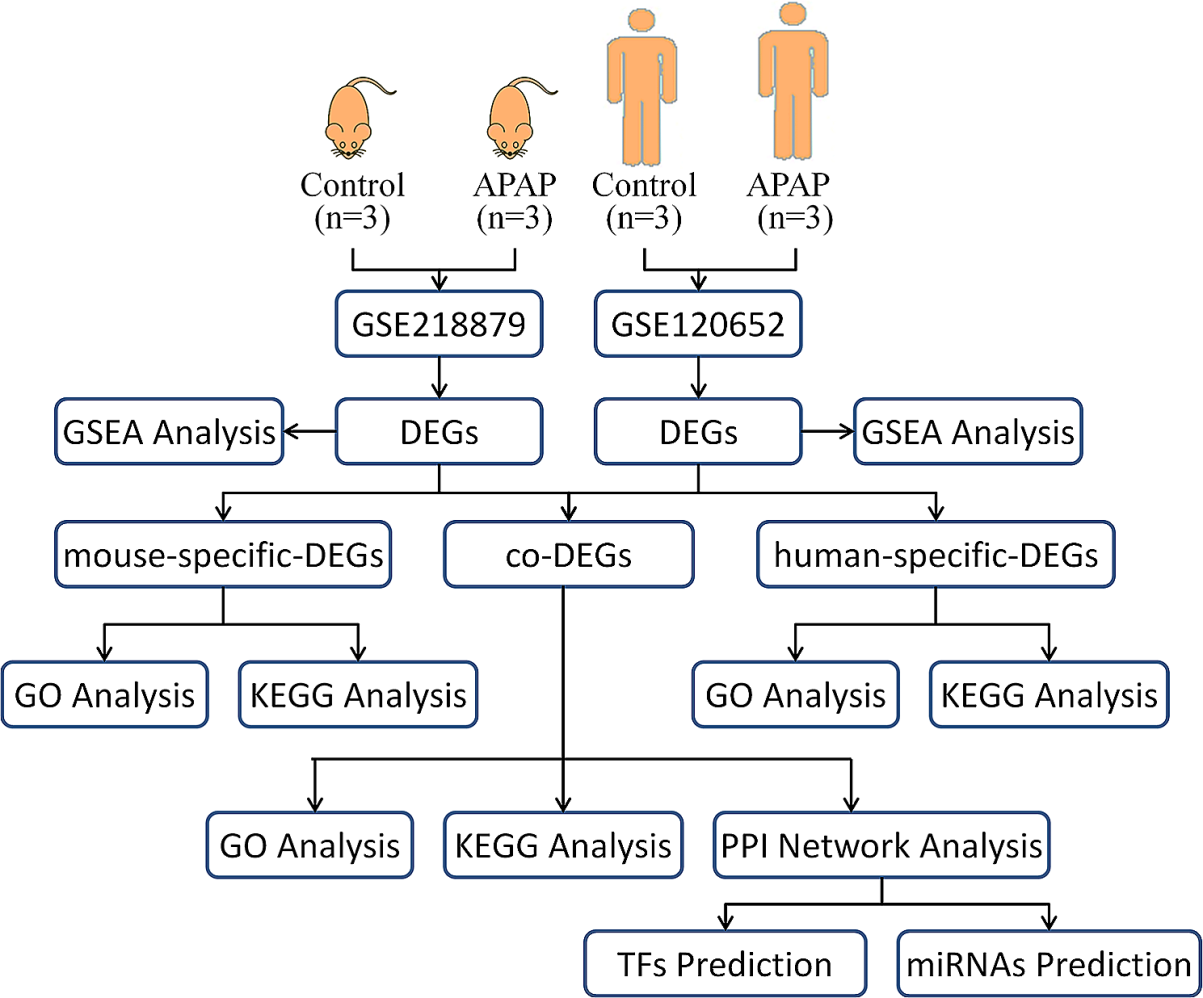
The flow chart of this study. DEGs = differentially expressed genes; co-DEGs = co-expressed DEGs; specific-DEGs = specific-expressed-DEGs; GSEA = Gene Set Enrichment Analysis; GO = Gene Ontology; KEGG = Kyoto Encyclopedia of Genes and Genomes; PPI = Protein-protein interaction; TFs = transcriptional factors; miRNAs = microRNAs

Fig. 1 shows the overall design and flow diagram of this study. Data from the two platforms were analyzed separately, differentially expressed genes identified from each platform were then intersected to identify genes that were both common and differentially expressed. The R package “Limma” was used to identify DEGs among AILI and healthy groups in the two datasets [14]. Pheatmap and ggplot2 were used to represent the DEGs with an absolute log2 fold change (logFC) > 0.5 and adjusted *P* value of 0.05. Then mouse gene names were converted to human gene names with R packages “biomaRt”. R package“ggvenn” was used for screening co-DEGs of the two datasets.

### Differential gene enrichment analysis

Based on DEGs, Gene Ontology (GO), Kyoto Encyclopedia of Genes and Genomes (KEGG) [15], and Gene Set Enrichment Analysis (GSEA) analyses were performed using the R package “clusterProfiler” (version 4.2.2). The *p*-value < 0.05 was considered to indicate statistical significance.

### Protein-protein interaction (PPI) network construction

PPI networks of co-DEGs were constructed using STRING (https://string-db.org/) and visualized using Cytoscape (version 3.9.1). CytoHubba (version 0.1), a plug-in of Cytoscape, was used to filter the top 20 genes using three algorithms, including MCC, MNC and Degree. The Venn diagram of these hub genes was gathered using R package “ggvenn”. Based on the PPI network, the most closely connected modules were chosen for further analysis using Cytoscape’s MCODE plug-in (version 2.0.2).

### TF-gene network and gene-miRNA interactions network of hub genes

Based on the JASPAR database, we constructed a network involving transcriptional factors (TFs) and hub genes through NetworkAnalyst (https://www.networkanalyst.ca) to understand how hub genes are potentially regulated. Furthermore, the Tarbase and miRTarBase v8.0 database were utilized to construct the gene-miRNA interactions network to predict the correlation between hub genes and miRNAs using the minimum netwok analysis. Degree ≥ 1 was used as the cut-off criterion for the results.

## Results

### Identification of DEGs from mice and humans samples

**Fig. 2:**
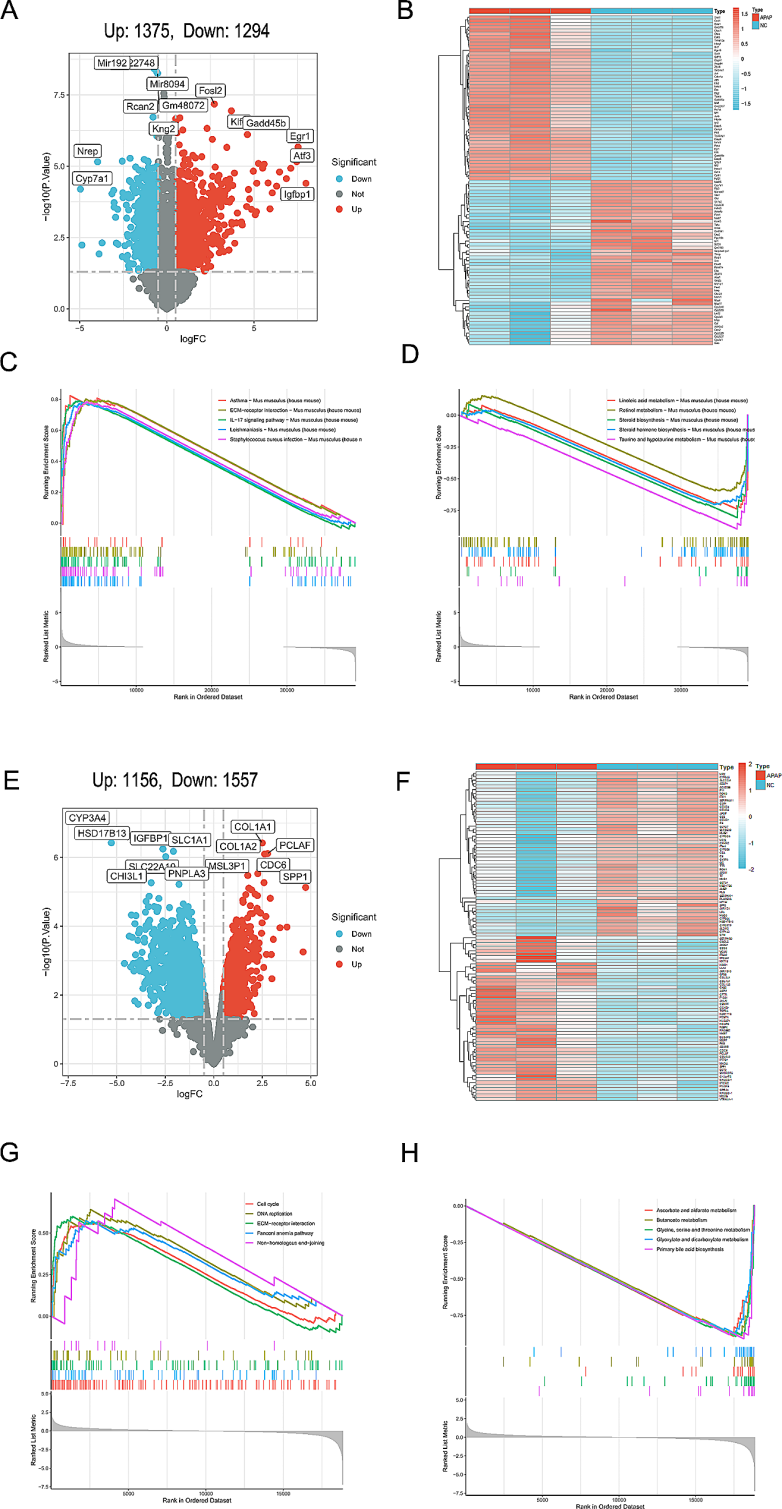
Identification of DEGs from GEO datasets. (**A**) The volcano map of GSE218879; (**B**) Heatmap of the GSE218879 dataset; (**C**,**D**) GSEA analysis of DEGs in GSE218879; (**E**) The volcano map of GSE120652; (**F**) Heatmap of the GSE120652 dataset; (**G**,**H**) GSEA analysis of DEGs in GSE120652. GSEA displayed only the top 5 results of each group

A total of 2669 DEGs were identified from the dataset GSE218879, which contained 1375 up-regulated genes and 1294 down-regulated genes (Fig. 2A and B, Supplementary Table S1). The GSEA results showed that there were a total of 113 gene sets significantly enriched (*p*-value < 0.05). Fig. 2C showed upregulation of the top 5 KEGG pathway enrichment in AILI group and control group, including asthma, ECM-receptor interaction, IL-17 signaling pathway, *Staphylococcus aureus* infection and *Leishmaniasis*. Fig. 2D showed downregulation of the top 5 KEGG pathway enrichment, including taurine and hypotaurine metabolism, steroid biosynthesis, linoleic acid metabolism, steroid hormone biosynthesis and retinol metabolism.

**Fig. 3:**
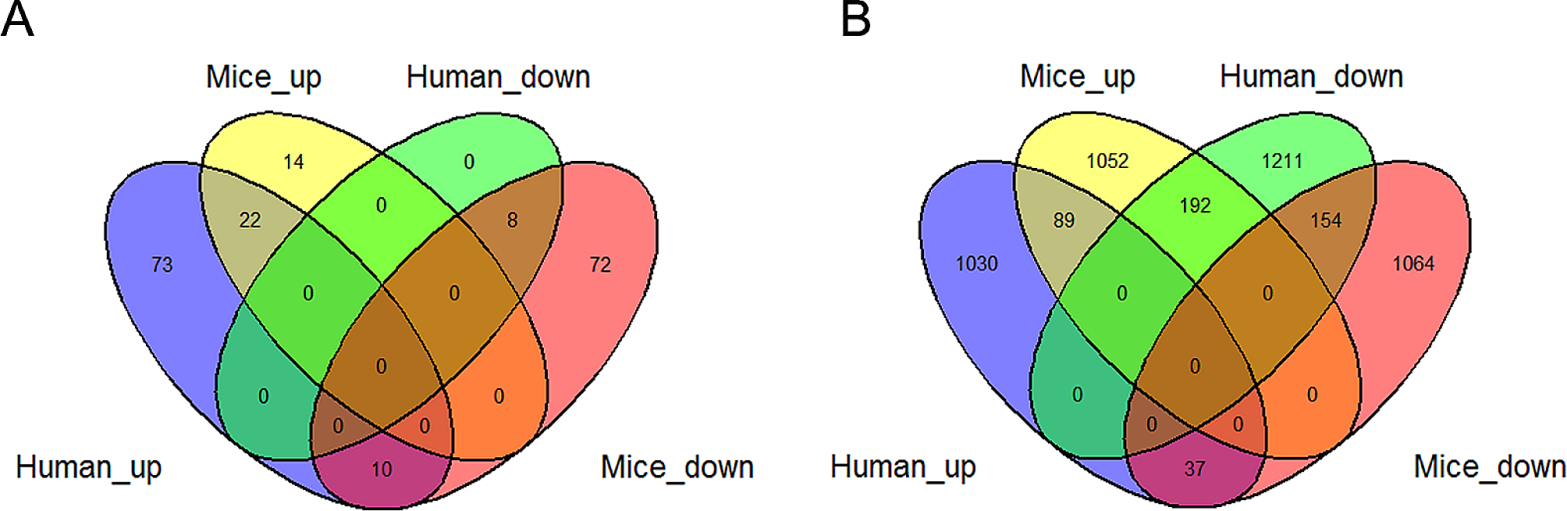
Differentially expressed genes. (**A**) Venn diagram of the GSEA-identified gene sets in GSE218879 and GSE120652. (**B**) Venn diagram of the intersection of up-regulated and down-regulated differentially expressed genes in GSE218879 and GSE120652

The dataset GSE120652 contained 1156 genes with up-regulations and 1557 genes with down-regulations (Fig. 2E and F, Supplementary Table S2). The GSEA results showed that there were a total of 126 gene sets significantly enriched (*p*-value < 0.05). Fig. 2G showed upregulation of the top 5 KEGG pathway enrichment in ALF group and control group, including non-homologous end-joining, DNA replication, ECM-receptor interaction, fanconi anemia pathway and cell cycle. Fig. 2H showed downregulation of the top 5 KEGG pathway enrichment, including primary bile acid biosynthesis, glycine, ascorbate and aldarate metabolism, butanoate metabolism, glyoxylate and dicarboxylate metabolism. As showed in Fig. 3A, there were 30 common gene sets simultaneously up-regulated or down-regulated between mice and humans. The comparison showed that 4 of the top 5 KEGG enrichment pathways up-regulated in mice were also significantly enriched in humans, and the top 5 KEGG enrichment pathways down-regulated in mice were also significantly enriched in humans.

### DEGs between mice and humans samples

A Venn map using co-downregulated and co-upregulated DEGs from GSE218879 and GSE120652 datasets was created based on converted mouse genes into human genes (Fig. 3B). Furthermore, 154 co-downregulated DEGs and 89 co-upregulated DEGs were identified (Supplementary Table S3). A total of 1052 up-regulated genes and 1064 down-regulated genes were only present in GSE218879, which were named mouse-specific-DEGs. 1030 up-regulated and 1211 down-regulated were only observed in GSE120652, which were named human-specific-DEGs. 192 genes showed upregulation in mice, but downregulation in humans. 37 genes showed downregulation in mice, but upregulation in humans. Therefore, genes with a significant difference in variance differed greatly between mouse and human liver in AILI.

### Gene enrichment analysis of specific-DEGs

**Fig. 4:**
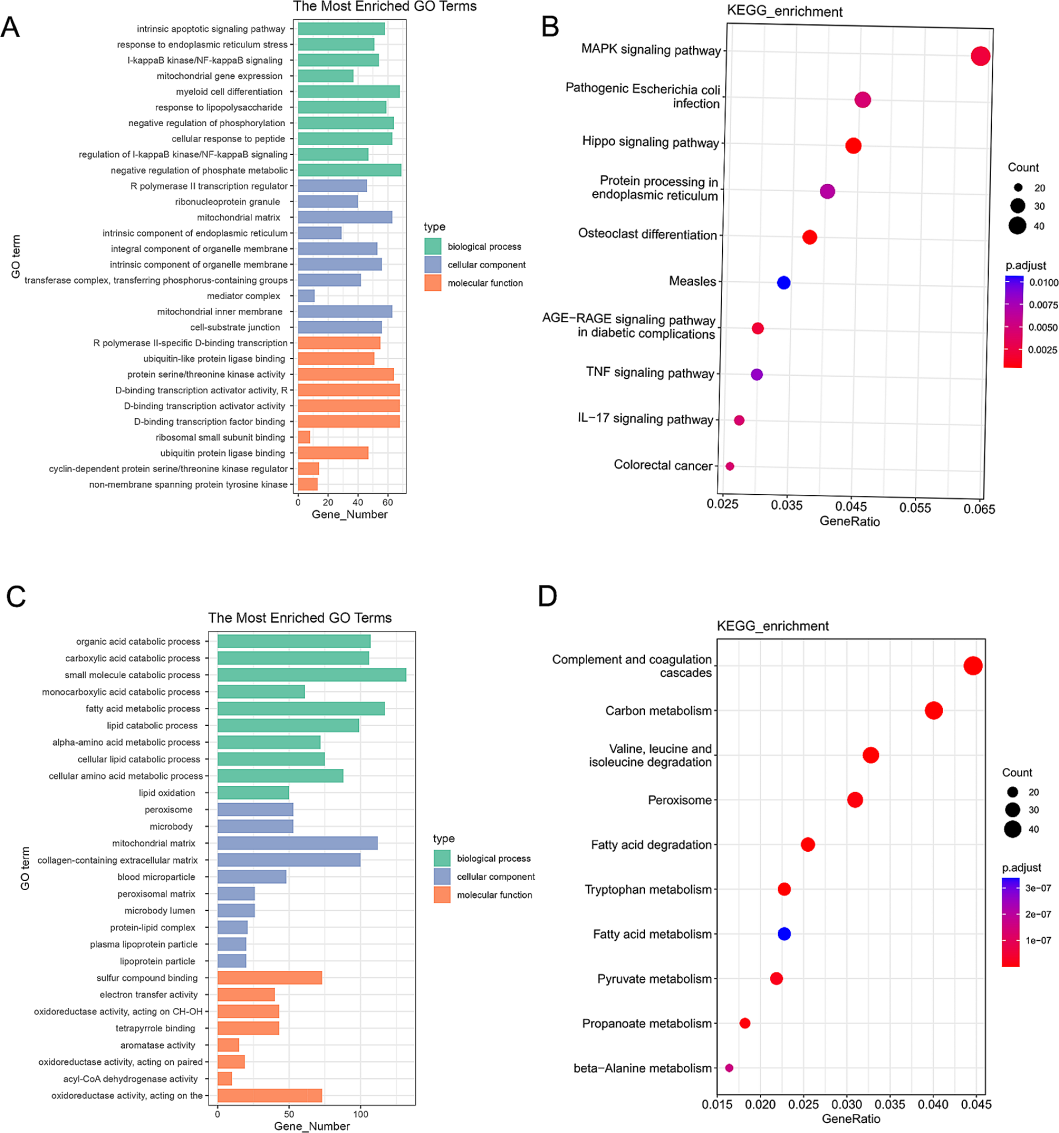
Gene enrichment analysis of specific-DEGs. (**A**,**B**) GO/KEGG enrichment analyses of mouse-specific-DEGs; (**C**,**D**) GO/KEGG enrichment analyses of human-specific-DEGs

Furthermore, we performed the functional enrichment of mouse-specific-DEGs and human-specific-DEGs via GO and KEGG. A total of 344 gene functions were significantly enriched from mouse-specific-DEGs (*p* < 0.05), including 314 biological processes (BP), 14 molecular functions (MF) and 16 cellular components (CC). 568 BP related items, 86 CC related items, 135 MF related items of human-specific-DEGs were screened out (*p*-value < 0.05). GO analysis in Fig. 4A revealed that mouse-specific-DEGs predominantly engaged in processes related to apoptosis and endoplasmic reticulum stress, and the enrichment of cell components mainly involved nucleus-related molecules. KEGG pathway analysis in Fig. 4B showed that the MAPK signaling pathway was the most abundant pathways. While the main biological processes of human-specific-DEGS were concentrated around catabolic process (Fig. 4C). Meanwhile, complement and coagulation cascades were the significantly changed pathways, as determined by KEGG analysis (Fig. 4D).

### Gene enrichment analysis of co-DEGS

**Fig. 5:**
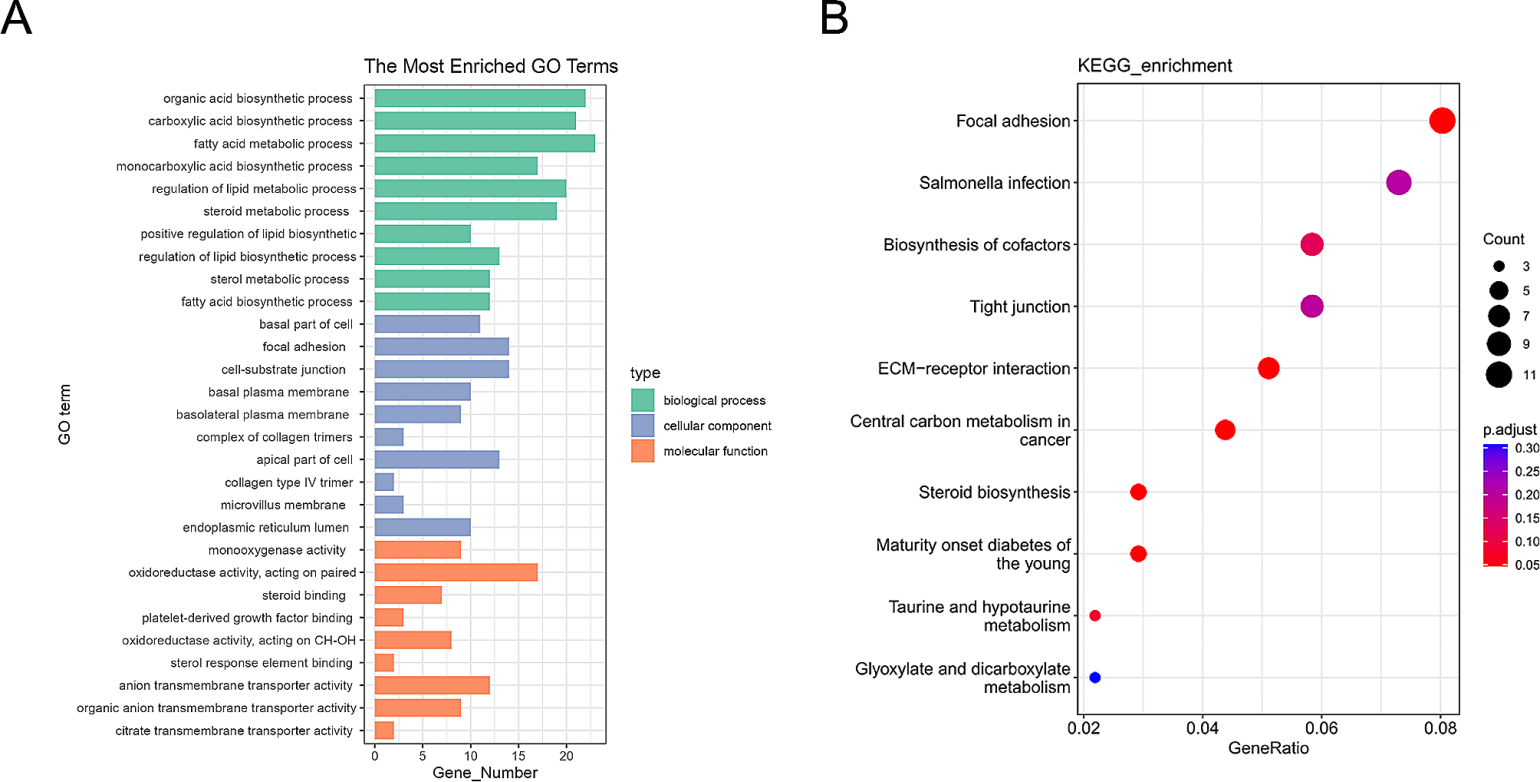
Gene enrichment analysis of co-DEGs. (**A**) GO/KEGG enrichment analyses of co-DEGs; (**B**) KEGG enrichment analyses of co-DEGs

The co-DEGS of mice and humans could be more valuable for studying human diseases, so we analyzed the role of co-DEGs in ALF by GO and KEGG. There were 568 BP related items, 86 CC related items and 135 MF related items of human-specific-DEGs were highly enriched (*p*-value < 0.05) for co-DEGs. GO BP showed that they were mainly enriched in biosynthetic and metabolism-related processes, including organic acid biosynthetic process, carboxylic acid biosynthetic process, fatty acid metabolic process, monocarboxylic acid biosynthetic process and regulation of lipid metabolic process (Fig. 5A). Enrichment analyses of the co-DEGs identified 23 KEGG pathways (*p*-value < 0.05). As shown in Fig. 5B, the top 10 KEGG terms are associated with the co-DEGs. KEGG pathway annotation showed that steroid biosynthesis, focal adhesion, ECM-receptor interaction, maturity onset diabetes of the young, central carbon metabolism in cancer, taurine and hypotaurine metabolism, biosynthesis of cofactors, tight junction and *salmonella* infection were the significantly changed pathways.

### Screening hub genes by PPI networks

**Fig. 6:**
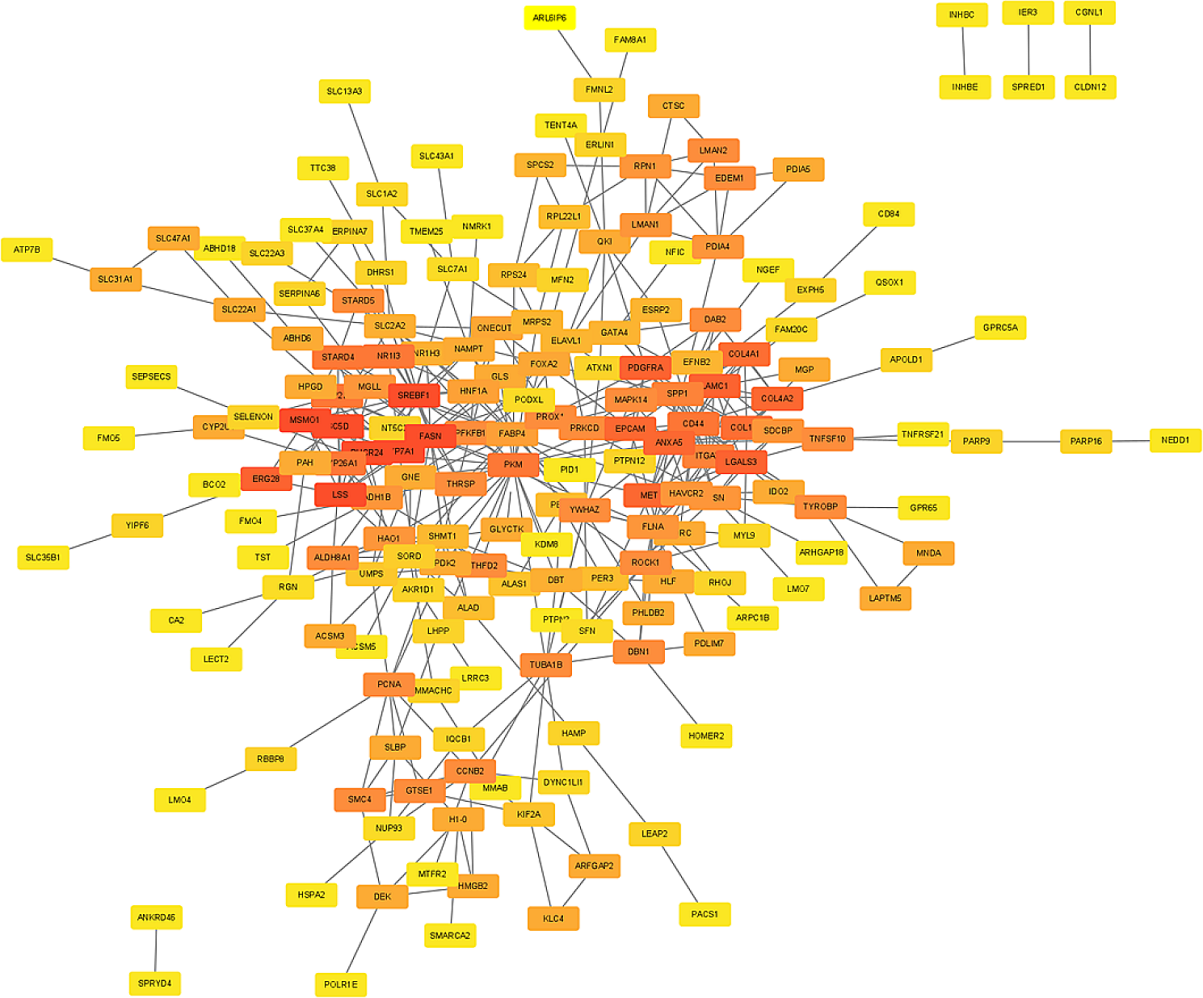
PPI network diagram of co-DEGs. The network diagram contains 193 nodes and 440 edges

**Fig. 7:**
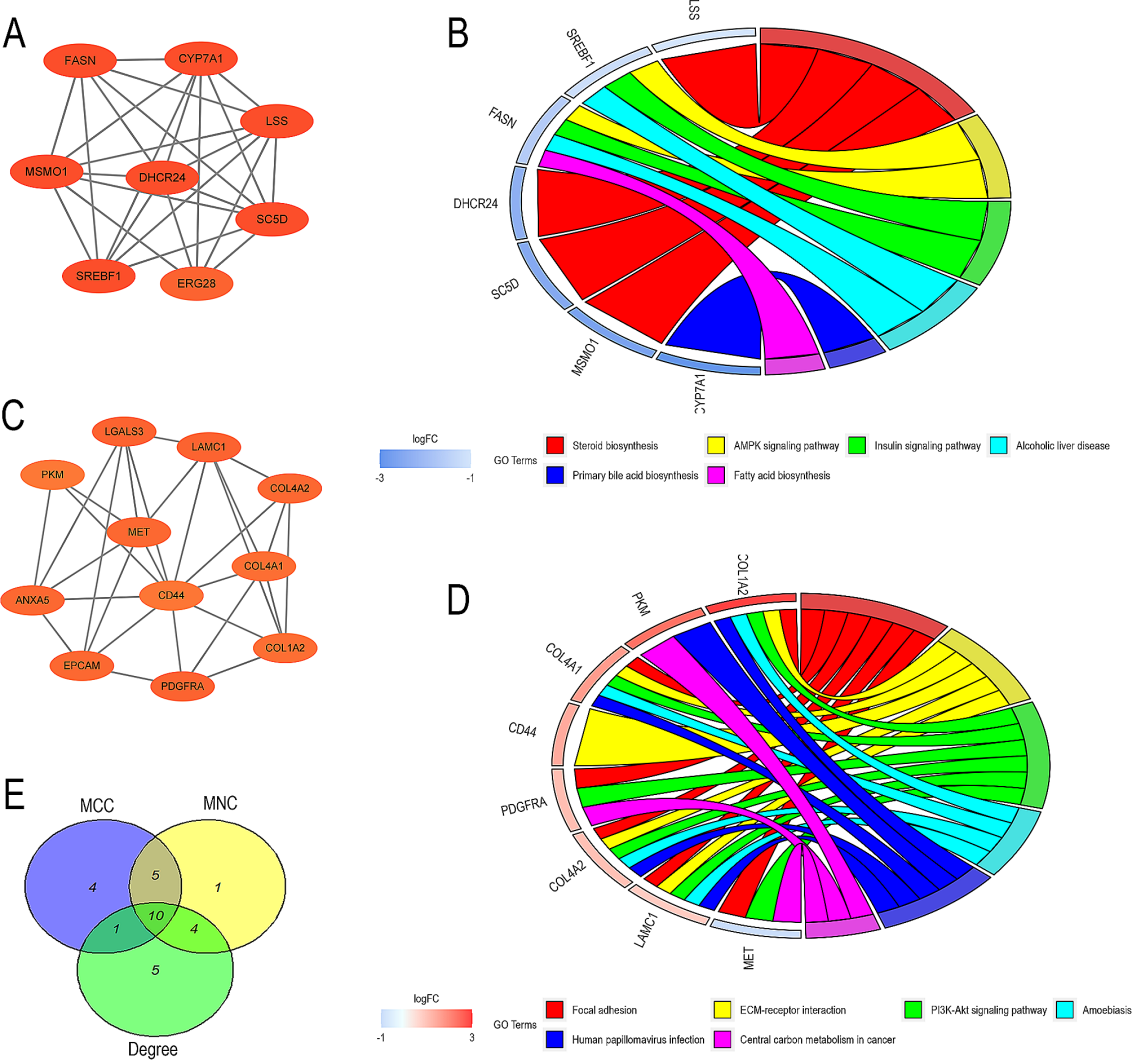
The critical modules of the PPI network diagram. (**A**) Key modules 1 of PPI network. (**B**) The chord diagram of modules 1 KEGG analysis. (**C**) Key modules 2 of PPI network. (**D**) The chord diagram of modules 2 KEGG analysis. (**E**) A Venn diagram showed that ten hub genes were identified

With the String database, co-DEG networks were created and then mapped using Cytoscape (Fig. 6). In the PPI network diagram, there are 193 nodes and 440 edges. The MCODE plug-in analyzes the critical modules, and two modules with the highest score are shown in Fig. 7A and C. In KEGG analysis, the first module was mostly clustered in steroid biosynthesis, AMPK signaling pathway, insulin signaling pathway, alcoholic liver disease signaling pathway, while the second module was mostly clustered in focal adhesion, ECM-receptor interaction, PI3K-Akt signaling pathway, amoebiasis, *Human papillomavirus* infection, central carbon metabolism in cancer (Fig. 7B and D). We ranked the top 20 genes of the whole network based on the Cytoscape plugin CytoHubba models: MCC, MNC and Degree. After taking the intersection of these three data sets, 10 hub genes containing *CYP7A1, LSS, SREBF1, FASN, CD44, SPP1, ITGAV, ANXA5, LGALS3* and *PDGFRA* were collected for further study (Fig. 7E; Table 1).

**Table 1:** A total of 10 hub genes screened by MCC, MNC and Degree methods, and showed the functions by literature search

Name	Express by bioinformatics analysis	Function
CYP7A1	down	Catalyze a rate-limiting step in cholesterol catabolism and bile acid biosynthesis [29].
LSS	down	Synthesize lanosterol in the key cyclization reaction of l cholesterol biosynthesis and associated with glutathione peroxidase activity (Gpx) levels [30].
SREBF1	down	Regulate transcription of the LDL receptor gene as well as the fatty acid and to a lesser degree the cholesterol synthesis pathway [31].
FASN	down	Catalyze the formation of long- chain fatty acids from acetyl-CoA, malonyl-CoA and NADPH [32].
CD44	up	The Chi3l1/CD44 axis as a critical pathway mediating APAP-induced hepatic platelet recruitment and tissue injury [33].
SPP1	up	Characterize bile duct–associated macrophages and correlates with liver fibrosis severity [35, 36].
ITGAV	up	Play a central role in the progression of liver fibrosis as a known profibrogenic cytokine [38].
ANXA5	up	Regulate hepatic macrophage polarization [34].
LGALS3	up	Act as a mediator of acute inflammatory responses, including neutrophil activation and adhesion, monocyte macrophage hemoattraction, etc. [37].
PDGFRA	up	Play an essential role in the regulation of embryonic development, cell proliferation, survival and chemotaxis [39].

### TF-gene network and gene-miRNA interactions network

**Fig. 8:**
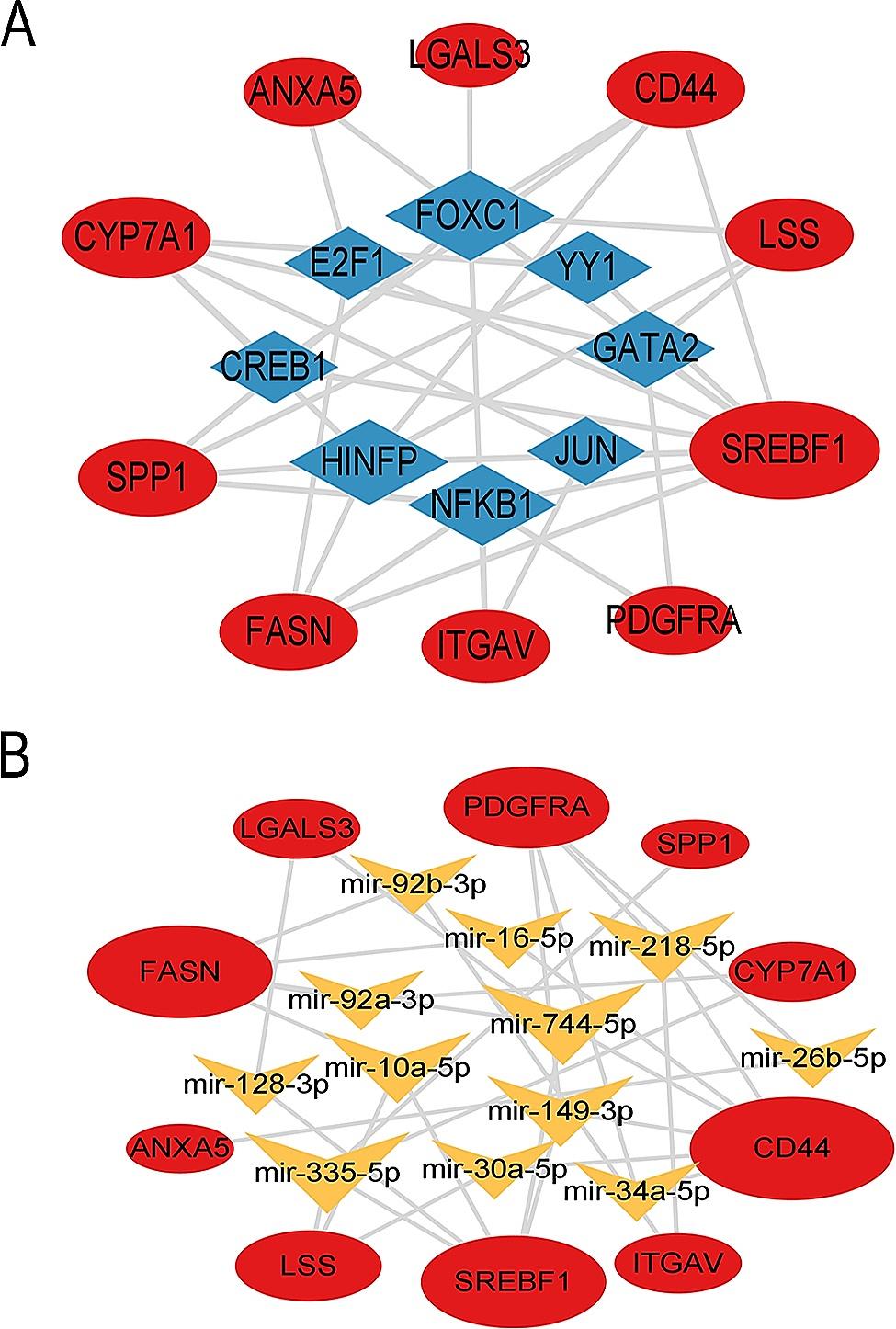
TF-gene and gene-miRNA interactions network. (**A**) Network for TF-gene interaction with the hub genes. 3 transcription factors with degree ≥ 5, including *FOXC1*, *HINFP* and *NFKB1*. (**B**) Minimum order PPI network constructed for the hub genes. 5 miRNA with degree ≥ 3, including mir-744-5p, mir-335-5p, mir-149-3p, mir-218-5p and mir-10a-5p

Our TF–hub gene regulatory network (Fig. 8A) was constructed using 8 TFs predicted by the JASPAR database to interact with 10 hub genes. We found 3 transcription factors with degree ≥ 5, including *FOXC1* (degree: 7, betweenness: 36.87), *HINFP* (degree: 6, betweenness: 13.1) and *NFKB1* (degree: 5, betweenness: 14.77), which may guide us to further mechanistic research. We used the mirTarBase v8.0 to construct the gene-miRNAs interactions network, which containing 26 nodes and 40 edges (Fig. 8B). It showed that mir-744-5p (degree:4, betweenness:37.98), mir-335-5p (degree:4, betweenness:36.47), mir-149-3p (degree:3, betweenness:38.74), mir-218-5p (degree:3, betweenness:22.44) and mir-10a-5p (degree:3, betweenness:15.05) interact most intensively with hub genes.

## Discussion

APAP overdose is a common cause of DILI. In spite of significant progress in understanding DILI, APAP hepatotoxicity remains poorly understood [16]. Further more, standard criteria for diagnosis and effective management for APAP-induced liver injury (AILI) are not established yet [17]. Hence, further understanding of AILI mechanisms is crucial. The use of animal models for investigating AILI is an important step in preclinical research. The most common models used to study APAP hepatotoxicity are rodents, including mice and rats [11, 18]. Mice are the preferred animal for studies of APAP overdose [19]. But there are significant biological variations and metabolic differences between humans and mice [10], which have severely hampered to unravel hidden mechanisms that occur in vivo.

The GSEA results showed most signal pathways do not overlap between 113 gene sets significantly enriched in mice and 126 gene sets significantly enriched in human. It confirms that mice and humans have substantially different biology and metabolism, as well as pharmacokinetics and toxicity profiles of drugs [20]. There were 30 identical signaling pathways, some of which have been proven to function in liver injury in mice and humans. For example, the IL17 signaling pathway is a common up-regulated signaling pathway in two species, which has been validated in both mouse and human samples [21–24]. According to evolutionary theory, mice and humans must have had some differences, which should be investigated by molecular analysis of their organs [25–27]. In order to further analyze the difference in molecular mechanisms, we transformed mouse genes conversion into human homologous genes and compare the differentially expressed genes in mice and humans. Among over 2500 DEGs examined, 243 genes were consistently up-regulated or down-regulated in both species, with a proportion of less than 10%. It appeared that orthologous gene levels were quite different, which may indicate that each species has a unique regulatory function. GO analysis showed mouse-specific-DEGs predominantly engaged in processes related to apoptosis and endoplasmic reticulum stress, but human-specific-DEGs were concentrated around catabolic process. It suggests that mice may have stronger catabolic capacity than humans, as reported by Straniero et al. [28] that mice has higher cholesterol synthesis and faster clearance of bile acids (BAs) than humans. Therefore, it could be inferred that there is a significant difference in the physiological processes triggered by APAP in the liver of two species. The species differences in the mechanisms of drug toxicity between mice and humans should be given sufficient attention.

In this study, we focused our analysis on the co-DEGs of mice and humans that may be more valuable for studying human diseases. GO BP showed that they were mainly enriched in biosynthetic and metabolism-related processes, including organic acid biosynthetic process, carboxylic acid biosynthetic process, fatty acid metabolic process, monocarboxylic acid biosynthetic process and regulation of lipid metabolic process. KEGG pathway annotation showed that steroid biosynthesis, focal adhesion, ECM-receptor interaction, maturity onset diabetes of the young, central carbon metabolism in cancer, taurine and hypotaurine metabolism, biosynthesis of cofactors, tight junction and *Salmonella* infection were the significantly changed pathways. These identical signaling pathways may be more helpful in guiding us to further mechanistic research of human ALF using mouse models.

The 10 hub genes were *CYP7A1, LSS, SREBF1, FASN, CD44, SPP1, ITGAV, ANXA5, LGALS3* and *PDGFRA*. Among these genes, the first four are down-regulated and the last six are up-regulated. In order to verify the function of these genes in ALF, relevant literature was searched and summarized in Table 1. We found that *CYP7A1*, *LSS*, *SREBF1*, *FASN* are down-regulated and mainly participate in the synthesis of cholesterol in two species [29–32]. This suggests that cell damage caused by APAP is reflected in the obstruction of cholesterol synthesis in liver cells. *CD44* is the up-regulated gene with the highest score using three algorithms, but research on its role in liver injury is scarcely presently. Zhao et al. [33]found that *CD44* knockout mice developed attenuated AILI with markedly reduced hepatic platelet accumulation. It suggests that the significant increase in *CD44* expression in mouse and human livers may be an important mechanism mediating liver injury. *ANXA5*, *SPP1* and *LGALS3* have been found to be involved in the regulation of the inflammatory response within the liver. A study shows that *ANXA5* regulates hepatic macrophage polarization [34]. De et al. [35, 36] confirmed that MoMFs lacking *SPP1* were more inflammatory in vitro and in vivo. The livers of mice lacking *LGALS3* showed a higher number of CD11b(+)/Ly6C(lo) macrophages after APAP treatment [37]. *ITGAV* and *PDGFRA* are two genes up-regulated, which could promote hepatic fibrosis [38, 39]. It may be more advantageous for us to use mouse models to reveal the mechanism of human liver injury by utilizing these hub genes that intersect with humans and mice. The TFs-genes network showed that *FOXC1*, *HINFP* and *NFKB1* may be the key regulatory factors of hub genes. *FOXC1* was able to induce liver repair after bile-duct-ligation-induced liver injury [40]. *HINFP*, a histone cell cycle regulator, could control cell cycle depending on the cellular context [41]. *NFKB1*, a member of NF-κB family, have been shown to play critical regulatory activities of inflammatory responses [42].

Overall, this study represents the first attempt at comparison of gene expression induced by APAP between mouse and human. The species-specific genes and co-expressed genes were identified from two species. But our study has several limitations. Firstly, we derived our conclusions from the public database and a small sample size. It is necessary to compare the results of basic and clinical research to verify the robustness of the results. Secondly, the severity of AILI was affected by factors such as age, ethnicity, gender, nutritional status, etc. [43, 44]. This could exacerbate the complexity of revealing mechanisms of drug-induced injury and affect the consistency of research research findings. Lastly, additional experimental confirmation is needed to further corroborate the function of hub genes and signaling pathways in AILI.

## Conclusion

In summary, the differential expression of genes and signaling pathways in AILI patients and mouse models was analyzed. The hub genes (*CYP7A1, LSS, SREBF1, FASN, CD44, SPP1, ITGAV, ANXA5, LGALS3, PDGFRA*) and pathways (steroid biosynthesis, ECM-receptor interaction, focal adhesion, etc.) identified could provide a more powerful reference for the study of human APAP-induced related mechanisms and therapeutic targets using mice as models.

### Electronic supplementary material

Below is the link to the electronic supplementary material.

Supplementary Material 1

Supplementary Material 2

Supplementary Material 3

Supplementary Material 4

## Data Availability

Data was collected from the GEO database (accession number GSE218879 and GSE120652). (https://www.ncbi.nlm.nih.gov/geo/).
