# Supplementary material for: Comparative analysis of gene expression between mice and humans in acetaminophen-induced liver injury by integrating bioinformatics analysis
Source: BMC Med Genomics. 2024 Mar 28;17:80. doi: 10.1186/s12920-024-01848-0 (PMC10976682; doi:10.1186/s12920-024-01848-0)
Supplement: Supplementary file 4 — Supplementary Material 4 [file 12920_2024_1848_MOESM4_ESM.docx]

**Supplementary Table Legends**

**Supplementary Table S1.** DEGs in mice samples.

**Supplementary Table S2.** DEGs in humans samples.

**Supplementary Table S3.** Co-expressed DEGs between mice and humans samples.
